# Novice Readers: The Role of Focused, Selective, Distributed and Alternating Attention at the First Year of the Academic Curriculum

**DOI:** 10.1177/2041669517718557

**Published:** 2017-07-07

**Authors:** Elena Commodari

**Affiliations:** Universita degli Studi di Catania, Catania, Italy

**Keywords:** reading acquisition, selective attention, focused attention, distributed attention, alternating attention

## Abstract

The ability to read depends on different cognitive skills. This study investigated the role of the main components of attention (selective attention, focused attention, distributed attention, and alternating attention) on the different dimensions of reading skills in novice readers. Participants were 288 Italian children, who attended the first year of primary school. Attention and reading skills (reading “comprehension,” “accuracy,” and “speed”) were measured. Different components of attention influence each dimension of reading. Moreover, both the correctness and rapidity at which attention operates play a pivotal role in learning to read. Interestingly, selective attention is involved in all dimensions of reading. These findings may have educational and practical relevance. The early assessment of attention might favor the development of new strategies of intervention in dyslexic children and in children at risk of developing learning difficulties.

Reading is the process of extracting and constructing meaning from written text for some purpose (Vellutino, Fletcher, Snowling, & Scanlon, 2004). It consists of the decoding of written signs that correspond to the phonetic unit of the language, identification of words, and reconstruction of the meaning of words and groups of words that compose a phrase. Reading skill comprises three dimensions: “comprehension,” which is the ability to understand words and text; “accuracy,” which is the ability to read text accurately, and “speed,” which is the ability to decode the written signs quickly.

Learning to read requires general cognitive functions, such as memory and attention (Baddeley, 1982; Savage, Cornish, Manly, & Hollis, 2006), and several specific abilities, such as linguistic and phonological skills (Bar-Kochva, 2013; van den Boer, van Bergen, & de Jong, 2014; Vellutino, Tunmer, Jaccard, & Chen, 2007). Several studies focused on the role of phonological skills and other underlying skills on reading acquisition and performance (e.g., Bishop & Snowling, 2004; Castles & Coltheart, 2004; Miller & Kupfermann, 2009; Share, 1995, 2004). Indeed only a few researchers investigated the relationship between reading and attention in typically developing (e.g., Bosse & Valdois, 2009; Commodari & Guarnera, 2005) and dyslexic children (e.g., Franceschini, Gori, Ruffini, Pedrolli, & Facoetti, 2012; Ruffino et al., 2010), although attention skills are damaged in several conditions, such as learning difficulties (Commodari & Di Blasi, 2014) or specific language impairment (Guarnera, Commodari, & Peluso, 2013).

## Attention Skills in Childhood

Attention is a complex function which participates in achieving and maintaining a state of alertness, orienting toward and selecting sensory events, and regulating thoughts and responses in an effortful goal-directed mode (Peterson & Posner, 2012; Pozuelos, Paz-Alonso, Castillo, Fuentes, & Rueda, 2014; Rueda, Posner, & Rothbart, 2005). Aside from the general cognitive functions, which are involved in reading, attention plays a pivotal role (La Berge & Samuels, 1974; Samuels, 1987).

Attention depends on three independent, but strictly related, networks: the “orienting,” “alerting,” and “executive” networks (Peterson & Posner, 2012). The “alerting network” is involved in the ability to prepare and sustain alertness to process high priority signals (Peterson & Posner, 1990). It is related to sustained vigilance (Posner, 2012) and contributes to increasing readiness to respond to impending stimuli (Yang & Yang, 2016). The “orienting network” is involved in the ability to prioritize sensory input by selecting a modality or location. It is responsible for the movement of attention through space to attend to sensory events (Posner, Rothbart, Sheese, &Voelker, 2014) and is entailed in target detection, selection of information, and engagement and disengagement of attention during attentional shifting (Posner, 2012). The “executive network” (Posner, 2012) plays a role in resolving a conflict among responses. It comprises several processes implicated in the planning and execution of goal-directed behaviors, such as anticipating consequences, selecting among competing stimuli, monitoring interruptions, or modifying behavior (Mezzacappa, 2004). Individuals differ in the development, efficiency, and variability of functioning within and across each of these networks (Mezzacappa, 2004). Key aspects of attention, such as focused attention, selective attention, divided attention, and alternating attention, depend on the functioning of these three networks (Posner, Petersen, Fox, & Raichle, 1988). In particular, “focused attention” depends on the alerting network, “selective attention” involves the executive network, “alternating attention” depends on the “orienting network” (Pozuelos et al., 2014), and “distributed attention,” also called “divided attention,” requires the functioning of each of these networks.

“Focused attention” concerns the ability to respond discretely to specific stimuli. It plays a pivotal role in many cognitive functions, such as problem-solving and reasoning (Sohlberg & Mather, 1989). “Focused attention” is measured through recognition tasks. The recognition tasks are often modeled on the well-known “Continuous Performance” test. The ability to maintain a state of preparedness for imminent processing of information, which characterizes “focused attention,” develops during the life span until young adulthood (Rueda et al., 2004). Children are less efficient than adults in response to alerting cues and present longer reaction times in the speed of preparing to alerting cues and sustaining this preparation (Pozuelos et al., 2014). “Selective attention” is the ability to avoid distracting stimuli (Lezak, Howieson, Bigler, & Trane, 2015; Sohlberg & Mather, 1989). It concerns the capacity to inhibit interference of distracting stimuli and suppress inappropriate responses. “Selective attention” is studied by tasks that involve conflict between different dimensions of a target stimulus, as in the Stroop task (Posner, Rueda, & Kanske, 2007; Stroop, 1935). These tasks commonly measure the ability to resist interference by distracting stimuli. The Stroop test is one of the most used measures of selective attention and executive functions (Chan, Shum, Toulopoulou, & Chen, 2008).

Some studies (e.g., Rueda, 2014; Rueda, Posner, & Rothbart, 2005) found that “selective attention” increases during early childhood, from toddlerhood into the second decade of life. The ability to carry out conflict tasks increases significantly from 2 to 7 years old (Commodari, 2016; Gerardi-Caulton, 2000; Rueda et al., 2004). However, the results of studies on changes in selective attention during the life span are not homogeneous. Some authors suggest that children from 10 years of age did not present significant differences in the execution of tasks that require resistance to the interference of competing demands, compared with adults (Ridderinkhoff, van der Molen, Band, & Bashore, 1997; Rueda et al., 2004). Other studies (e.g., Bunge, Dudukovic, Thomason, Vaidya, & Gabrieli, 2002) showed that adults performed better than children both in the resistance to distracting stimuli and the ability to inhibit responses or behavior.

“Alternating attention” is the rapid shifting of the attentional focus, given the inability to process all available information in parallel (Parasuraman, 1998). It concerns the ability to alternatively shift focus and tasks (Lezak et al., 2015), and the ability to disengage and reengage the focus of attention in response to environmental stimuli.

Alternating attention is usually measured through multiple-choice tasks such as adjustments of the cancellation test (e.g., Cannavò, Conti, & Di Nuovo, 2016; Di Nuovo, 2006), or multiple-object tracking tests (e.g., Sears & Phylyshyn, 2000). These tests require shifting attention from one focus to another rapidly. In particular, cancellation tests have a long history in neuropsychological assessment (Pradhan & Nagendra, 2008). This type of test can vary widely in complexity. It is administered as a paper-and-pencil or computerized task and is used to evaluate a person's ability to visually search for an identifiable target and to either cancel or circle all such target items in an array.

The ability to shift attention is present from infancy (Colombo, 2001; Posner & Raichle, 1994) and develops with age (Akhtar & Enns, 1989; Brodeur, Trick, & Enns, 1997; Enns, 1990; Trick & Enns, 1998). Some studies on the reorientation of attention, in which cues are presented opposite to the location of the subsequent target, have found that the time to disengage from the location of the cue decreased with increasing age (Akhtar & Enns, 1989). Moreover, the studies that computed the orienting effect by comparing reaction times to valid and invalid cued target found a change of orienting skills until about late childhood (Schul, Townsend, & Stiles, 2003; Waszak, Li, & Hommel, 2010).“Distributed attention,” is the ability to maintain two or more attentional focuses simultaneously and concerns the allocation of resources between different sets of input (Hahn et al., 2008; Parasuraman, 1998). Attention can be divided between locations in space, between features of a single or of several objects, and between stimuli in one or several sensory modalities (Braun, 1998). This skill is usually measured through tests that require the execution of dual tasks. There is considerable variation in the types of tasks used in the studies on distributed attention. Salthouse, Fristoe, Uneweaver, and Coons (1995) have reported that the tasks ranged from two perceptual discriminations to sentence recall and picture recognition, and others. Often the tasks combined two stimuli of a different type in the same test, with the aim to evaluate the subject’s ability to vary the attentional focus and, therefore, to distribute attention to the management of many different stimuli (Cannavò et al., 2016).

Some authors (e.g., Dye & Bavelier, 2010; Trick & Enns, 1998) found that the ability to distribute attention increases with age. In particular, Dye and Bavelier (2010) found that the ability to distribute visual attention across the field to search for a target improves across the school-age years. Moreover, children who were able to play video games presented better ability to distribute attention. These authors found that when basic attentional skills were involved, children who were exposed to action-based games showed better performance, above and beyond that expected by maturational processes. However, it is not clear whether the effect of video games on the development of the various aspects of visual attention is causal or whether children who possessed better-than-average visual attention skills might be drawn toward playing action-based video games; thus, placing themselves within an environment that leads to further improving those visual skills.

## Why Attention Influences Reading Acquisition

La Berge and Samuels (1974) hypothesized a close relationship between attention and reading skills. They thought that reading difficulties are related to lack of automaticity in decoding, which overloads the attentional system (Samuels, 1987). From this model, several researchers studied attention engagement during reading (e.g., Bosse & Valdois, 2009; Commodari & Guarnera, 2005; Ruffino et al., 2010).

The research found that “focused attention” and “selective attention” influence accuracy in word identification and letter-sound decoding (Muter, Hulme, Snowling, & Stevenson, 2004). Moreover, “selective attention” and “alternating attention” are involved in the preparation of saccadic eyes movements (Hoffman & Subramaniam, 1995; Kristjánsson, 2007, 2011). In this regard, Hoffman and Subramiam (1995) suggested that the readers use spatial attention in the programming and execution of saccadic eye movements. In their studies, these authors found that visual-spatial attention is an important mechanism in generating voluntary saccadic eye movements.

“Alternating attention” is also implicated in reading performance. Some studies (e.g., Francis, Kaganovich, & Driscoll-Huber, 2008; Gordon, Eberhardt, & Rueckl, 1993) found that phonetic discrimination and speech signal segmentation during reading involved the rapid shifting of attention. An accurate and rapid attentional shifting contributes to the segmentation of a word into its graphemes. Others researchers (e.g., Reynolds & Besner, 2006; Shaywitz & Shaywitz, 2008) suggested that attention is critical for translating print into speech and that attention is crucial for fluent reading.

The studies on learning difficulties confirmed the role of attention in reading performance and showed that children with learning disabilities often present deficits in attention (Brook & Boaz, 2005; Tirosh, Cohen, Berger, Davidovitch, & Cohen-Ophir, 2001).

A visual attention span deficit is a well-documented factor that contributes to developmental dyslexia, independently of a phonological disorder (Bosse, Tainturier, & Valdois, 2007). Visual attention span, which reflects the number of orthographic units that can be processed in a glance (van den Boer et al., 2014), influences word reading speed and accuracy (Bosse & Valdois, 2009; Valdois, Bosse, & Tainturier, 2004; van den Boer, de Jong, & Haentjens-van Meeteren, 2013). In particular, children with dyslexia have deficiencies in the simultaneous processing of string elements. They have limitations in the number of string elements they were able to process simultaneously (Hawelka & Wimmer, 2005; Pammer, Lavis, Cooper, Hansen & Cornelissen, 2005; Pammer, Lavis, Hansen & Cornelissen, 2004; Pelli, Burns, Farell, & Moore-Page, 2006; Valdois, Bosse, & Tainturier, 2004). Moreover, preschoolers at risk of developing learning difficulties present higher response times in tasks measuring auditory selective attention, and a lower rate of accuracy on visual-spatial selectivity tasks, compared with their peers without learning difficulty risk (Commodari, 2012). In this regard, Franceschini et al. (2012), in a longitudinal study, found that visual-spatial attention in preschoolers specifically predicts future reading ability. They found that poor readers in Grade 1, already presented a deficit in serial visual search as well as spatial cueing facilitation when they were prereaders. These results agree with those obtained in a study by Ferretti, Mazzotti, and Brizzolara (2008). These authors found that visual scanning abilities in kindergarten were good predictors of reading acquisition. Difficulties in reading comprehension were also frequently found in children and adolescents with Attention-Deficit or Hyperactivity Disorder (Stern & Shalev, 2013).

Although there is abundant evidence that attention plays a pivotal role in reading at each age and each level of competency (e.g., Commodari & Guarnera, 2005; Vellutino et al., 2004), its engagement during reading activities depends on a child’s level of reading skill. According to the classical “Information Processing Theory” (La Berge & Samuels, 1974), novice readers use all of their attention in decoding letters and words, and, for this reason, they encounter difficulties in comprehending what they have decoded. The scanning of text and identification of a word requires high attentional engagement in novice readers, whereas these activities become automatic in expert readers.

Engagement of attention during reading also depends on the opacity-transparency of the alphabetic writing systems of the language that a child speaks. Transparent orthographies are characterized by one to one grapheme–phoneme correspondence. Opaque orthographies do not have this correspondence. In the opaque orthographies, more than one grapheme corresponds to the same phoneme and, conversely, each grapheme represents several phonemes.

Research showed that children who spoke languages with opaque orthographies, such as English, acquired reading more slowly compared with their peers speaking transparent languages, such as Italian (Defior, 2004; Seymour, Aro, & Erskine, 2003). In this regard, Luoni et al. (2015) suggested that learning to read requires a higher engagement of attention in opaque orthographies than transparent orthographies. This high involvement of attention could explain the comorbidity between reading disability and attentional disorders in populations whose language has opaque orthography, even if there are no data supporting this hypothesis.

## Research Aims

The overall goal of this study was to investigate the relationships among attention and reading skills in novice readers. Although research has broadly investigated the role of attention on reading skills, the relative contribution of different aspects of attention to reading performance was not evaluated extensively. The majority of the previous studies have analyzed the role of single aspects of attention, such as selectivity, on the single dimensions that comprise reading skills, such as comprehension (e.g., Stern & Shalev, 2013) or accuracy in the decoding of letters of words (e.g., Bosse & Valdois, 2009). Furthermore, the majority of these studies were conducted on children speaking opaque languages (Bosse & Valdois, 2009; Stern & Shalev, 2013; Vellutino et al., 2007).

This work aimed at overcoming some of these limitations. It seeks to provide a better understanding of the contribution of the most important aspects of attention on the three dimensions in which reading skills are articulated (comprehension, accuracy, and speed) in a sample of novice readers, who spoke a language with transparent orthography. “Focused attention,” “selective attention,” “distributed attention,” and “alternating attention” have been evaluated.

## Method

### Participants

Participants were 288 Italian children (144 boys and 144 girls, mean age: 6.2, standard deviation: .59), who attended the first grade of primary school. In Italy, children begin primary school from 5.5 to 6 years old and learn to read in the first grade. Participants attended three public schools located in a large town of Italy. The academic curricula were the same for each school. All children were native Italian speakers with normal or corrected-to-normal vision. Children were tested in March. In Italy, the school-year begins at mid-September and ends at mid-June.

The sample was recruited from the 366 children who attended first grade in the schools involved in the study. All the children in first grade were tested for reading skills, with the exceptions of the children with physical or psychological certified disabilities and children who were native speakers of other languages. According to some of the criteria derived by the quoted Italian standardization (Cornoldi & Colpo, 2011; Cornoldi, Colpo, & Gruppo, 1998), which permits the identification of children whose level of reading is inadequate for their age and academic grade, children were classified as good or poor readers.

The participants who presented reading comprehension and accuracy scores less than those corresponding to the 15th percentile were classified as poor readers. In particular, poor readers were the children who presented a reading performance that indicated a needed surveillance, because this was not sufficient, even if these children were not in an apparent condition of risk for reading difficulty, and children who showed reading performance inadequate for their scholastic grade and required immediate intervention).^1^ Children who presented comprehension and accuracy scores more than those corresponding to the 51 percentiles were classified as good readers (children who presented sufficient or optimal reading comprehension). Reading accuracy was measured through “error rate” scores, and high scores indicated low performance (see Measures section for details). Considering that children who participated in the study were very novice readers, the “speed” scores were not used to select the poor readers, because the slowness of reading is common in this stage of acquisition of reading skills.

Three hundred sixty-six children were tested for reading skills. Of these, 144 children classified as “poor readers” and an equal number of “good readers” were involved in the study. The 144 “good readers” were selected according to the following procedure: (a) the “poor” and “good” readers for each class group were matched for age and gender. Children were chronologically matched within 2 months; (b) for each class group, all of the “poor readers” and the same number of matched “good readers” were recruited. When the “good readers” in a class group were more numerous than the matched poor readers, the good readers were randomly selected. The remaining students were excluded from the successive measures.

### Measures

The study was conducted using standardized instruments to measure attention reading skills. The Cornoldi, Colpo, and Gruppo MT battery (1998; Cornoldi & Colpo, 2011), which comprised a “comprehension” and a “speed and accuracy” test, measured reading skills. This battery is largely standardized on the Italian population, and it is used during the diagnostic process of learning disability. The “comprehension” test (Cronbach’s alpha= .77) required that children read a text and responded to 15 questions that examined their comprehension level. The questions were in a multiple-choice format and used different words from those in the text that children read. The score was the number of correct answers, and a high score indicated good performance. Scores less than 8 (5th percentile) indicated a clear status of reading difficulty for the scholastic grade, and scores less than 9 (15th percentile) indicated a reading performance not sufficient and the need to pay attention for risk of reading difficulties. Scores more than 13 indicated an optimal reading performance for the academic grade (75th percentile), while scores from 10 to 13 indicated a sufficient performance. Children were invited to bring pencil and eraser on the desk. The children were told that they would be distributed a little book containing a story and some questions with the aim to see if they were able to understand what they read. The examiner explained to the children that, for each question, they had to choose the correct answer from four alternatives. To minimize the weight of the children's memory, they could reread the text as often as desired.

The “accuracy and speed” test consisted of a written paragraph, printed on a card. The child is required to read the paragraph as fast as possible, avoiding mistakes. The test has no time limits, but the time employed by the subject is recorded. Reading accuracy was measured through “error rate” scores, which were the sum of the errors made during the reading of the text (e.g., omissions, letter substitutions, and pauses for more than 5 s). “Speed” scores were calculated by considering the number of syllables that a child read in 1 s (Cornoldi, Tressoldi, & Perini, 2010). In particular, the “speed” scores were calculated using a transformation in seconds/syllable. A low score indicated good performance. “Error rate” scores from 4 to 5 (15th percentile) indicated a condition of risk for reading difficulty, scores more than 5 (5th percentile) indicated a clear condition of reading difficulties. “Speed” scores from .04 to .4 indicated a condition of risk for reading difficulties, while “speed” scores < .04 indicated a clear condition of reading difficulty (Cornoldi & Colpo, 2011; Cornoldi et al., 2010).

The reading battery has good psychometrics properties. Its reliability ranges from .75 to .89 for “accuracy” scores and from .94 to .96 for “speed” scores.

Several computerized tasks measured attention skills. For each task, the number of correct responses and the median response times in seconds was calculated. Three recognition tasks measured auditory, visual, and visual-spatial “focused attention”; a computerized version of the classic Stroop test measured “selective attention;” simultaneous visual and auditory detection tasks measured “distributed attention”; a multiple barrage task assessed “alternating attention.”

The “focused attention” tasks involve target recognition and were modeled on the “Continuous Performance” test, which has both visual and auditory versions, such as the Auditory Continuous Performance Test developed by Keith (1994). In particular, the “auditory focused attention” task (reliability values: 91; concurrent validity: *r* = .90) required the recognition of an auditory target among vocal distractors. Participants had to press a particular computer key following a vocal stimulus, using their favorite hand. Children were sitting in front of the monitor of a personal computer. To avoid excessive head motion, the screen were placed not much higher than the keyboard (not over the central unit of the computer, as usually happens, but right in front of the keyboard). This position was maintained for all the tasks. The stimuli were letters (vowels and consonants). There were 30 items, 9 of which were targets. The target was the letter “o.” The “visual focused attention” task (reliability values: 90; concurrent validity: *r* = .90) required the recognition of a visual target among a group of distractors (images of common objects) appearing in the sequence. Participants had to press a key when a visual target appeared on the screen of the computer. There were 30 items, 9 of which were targets. The target was a star.

The “visual-spatial focused attention” task (reliability values: 89; concurrent validity: *r* = .90) was a computerized version of the symbols barrage test. Participants had to delete one target in a set of 36 stimuli, which appeared in the display. The screen showed a set of 36 symbols, which were sequentially circled from left to right. The target symbols were 12. Subjects had to press a computer key when the target was circled. The “selective attention” task (reliability values: 90; concurrent validity: *r* = .92) was a computerized version of the Stroop test. It comprised two tasks. The first task was the baseline. Participants had to press a computer key of the same color as the character used to print a word that appeared on the screen. The second task was the interference task. It required subjects to name the color of the ink used to print a word describing a different color, for example, “Red,” written using blue ink. After 3 s of each response of the participant, a new input appeared on the screen. The final computerized score, which measures the ability to resist distraction, is automatically obtained by taking the difference of the response times on the first and second tasks.

The “distributed attention” task (reliability values: 91; concurrent validity: *r* = .92) was a simultaneous dual task. This task required the subject to recognize a visual target and an auditory target simultaneously. It involved the ability to distribute attention on two parallel tasks. Children had to press a particular computer key following a visual target and simultaneously press another key following a particular auditory target. The visual target was a star; visual stimuli used as distractors were common objects. The auditory target was the word “sun”; auditory distractors were common words.

The “alternating attention” task was a multiple barrage task. It was a computerized adjustment of a nonverbal cancellation test. The task required rapidly shifting of attention and continual change of attentional orientation. It comprised two subtasks (verbal and visual-spatial). Both the verbal and visual-spatial tasks required search and cancellation of targets. The targets were presented in eight sets of 90 stimuli. For each set of stimuli, there were three targets. The sequence of the group of targets changed in each set. The participants had to change their attentional focus with the aim of identifying all of the targets. In the first subtask (verbal task), the stimuli were letters, in the second subtask (visual-spatial task), the stimuli were small squares with a variously oriented code, as in the Toulouse-Pieron test. This task required continual shifting of attentional focus. The test–retest reliability for this task was .82 for the verbal subtask and .80 for the visual-spatial subtask. The internal consistency was *.*89 and .87 for these subtasks, respectively; concurrent validity was .86 and .87 for the corresponding subtasks.

### Procedures

A trained psychologist administered the tests in the schools, during school time. The reading “comprehension” test was administered during collective sessions in each classroom. The other tests were administered in individual sessions. The setting was a familiar room in the school, away from distracting noises. A training session preceded the administration of the attention tests. Institutional review approval and parental consent for each child were obtained.

## Results

Several statistical analyses were conducted. Correlation analyses between the attention and reading scores and multiple regression analyses, using the attention scores as the independent variables and each reading score as the dependent variable, were calculated for the entire sample. Moreover, with the aim to better investigate the contribution of the main components of attention on reading skills, differences in attention skills by poor and good readers were measured.

### Correlation Analysis

A correlation matrix was performed. Table 1 shows the Pearson Correlation Coefficients corrected for multiple comparisons. The Pearson Correlation matrix showed that the reading scores were correlated with several attention scores. The finding suggests a relationship between attention skills and learning to read. Interestingly, different aspects of attention were differently correlated to the three dimensions of reading (see Table 1). In particular, reading comprehension was correlated to the response times of the “focused attention” tasks (auditory focused attention: *r* = −.11, *p* < .001; visual focused attention: *r* = −. 21, *p* < .001; visual-spatial focused attention *r* = −.13, *p* < .005), “distributed attention” (*r* = −.17, *p* < .001), and “alternating attention” tasks (alternating attention—verbal: *r* = −.14, *p* < .005; alternating attention—spatial: *r* = −.20, *p* < .001). Of interest, the correlations were negative. Presumably, children who were less impulsive in their responses presented a better analysis of the text and a better comprehension. The ability to resist interfering stimuli, which was measured through the “selective attention” task, and spatial “focused attention” were found to be correlated to reading accuracy, while the response times during the execution of selective attention and alternating attention tasks were correlated to reading speed.

**Table 1.** Correlation Matrix for All the Measures.

	1	2	3	4	5	6	7	8	9	10	11	12	13	14	15	16	17	18
1.																		
2.	−.11*																	
3.	−.27**	.49**																
4.	−.38**	.39**	.67**															
5.	.11	−.36**	−.09	−.02														
6.	−.11*	−.01	−.02	−.02	−.34**													
7.	.05	−.06	−.06	−.04	.06	−.32**												
8.	−.21**	−.04	.00	−.07	−.08	.44**	−.29**											
9.	.18**	−.09	−.21**	−.14*	.29**	−.31**	.14*	−.18**										
10.	−.13*	. 12*	.08	.03	−.26**	.43**	−.17**	.42**	−.57**									
11.	−.09	−.12*	−.10	.03	.09	−.16**	21**	.007	.15**	−.15*								
12.	−.17**	.05	.02	−.03	−.17**	.49**	−.28**	.36**	−.31**	.37**	−.22**							
13.	−.01	.02	.04	.04	.30**	−.20**	.21**	−.03	.17**	−.15*	.20**	−.10						
14.	−.14*	.10	. 21**	. 13*	. 02	−.02	.03	.15*	−.07	.12*	.04	−.02	.34**					
15.	.06	−.07	−.06	−.06	.20**	−.16**	.17**	−.18**	.15**	−.22**	20**	−.18**	.36**	.15**				
16.	−.20**	.006	.21**	.28**	.08	.03	−.03	−.02	−.11*	.01	.16**	.09	.29**	.48**	.27**			
17.	.20**	−.21**	−.10	−.14*	.04	.05	−.05	−.02	−.03	.05	.02	.02	.08	−.02	.14*	.01		
18.	−.04	.08	.18**	.15**	−.09	.06	−.02	.02	−.17**	.09	.01	.09	.01	.03	.05	.03	−.02	

*Note.* 1. Comprehension; 2. Reading accuracy—error rate; 3. Reading accuracy—RTs; 4. Reading speed; 5. Auditory focused attention—correct; Auditory focused attention—RTs; 7. Visual focused attention—correct; 8. Visual focused attention—RTs; 9. Spatial focused attention—correct; 10. Spatial focused attention—RTs; 11. Distributed attention—correct; 12. Distributed attention—RTs; 13. Verbal alternating attention—correct; 14. Verbal alternating attention—RTs; 15. Spatial alternating attention—correct; 16. Spatial alternating attention—RTs; 17. Selective attention—correct; 18. Selective attention—RTs.

* Correlation is significant at the .05 level. **Correlation is significant at the .001 level.

### Regression Analyses

Several multiple regression analyses, using “entry forced” method, were calculated. These analyses were conducted with the aim to investigate whether the different aspects of attention (auditory, visual, and visual-spatial “focused attention,” “selective attention,” “distributed attention,” and “alternating attention”) were predictive of the dimensions of reading skill. “Reading comprehension,” “reading accuracy,” and “reading speed” scores were regressed on all the attention scores with control for “gender” also included in the regression (see Table 2).

**Table 2.** Multiple Regression Analyses Using the Attentional Scores and Gender as the Independents Variables and Reading “Comprehension”, “Accuracy,” and “Speed” as the Dependent Variable.

	Reading scores					
	Comprehension *F*=3.16**; *R* Square: .15		Accuracy *F*= 3.86; *R* square: .18		Speed *F*=4.06** *R* square: .14	
Variables	Standardized coefficient B	*t*	Standardized coefficient B	*t*	Standardized coefficient B	*t*
Gender	−.04	−.64	.01	.15	−.12	−1.95
Auditory focused attention						
Correct	−.00	−.08	−.23	−3.68**	.02	.38
RTs	.02	.27	−.09	−1.26	−.01	−.17
Visual focused attention						
Correct	.07	1.13	−.07	−1.22	−.06	−1.02
RTs	−.14	−1.92	−.15	−2.21*	−−.10	−1.46
Visual-spatial focused attention						
Correct	.12	1.70	.08	1.122	−.08	−1.21
RTs	.04	.49	.16	2.07*	.04	.52
Distributed attention						
Correct	−.09	−1.49	−.12	−2.06*	.03	.60
RTs	−.08	−1.24	.03	.46	−.08	−1.20
Selective attention						
Correct	.19	3.28**	−.20	−3.57**	−.12	−2.22
RTs	.01	.17	.04	.84	.13	2.44*
Alternating attention (verbal)						
Correct	−.01	−.19	.18	2.75*	.03	.58
RTs	−.01	−.27	.07	1.03	−.002	−.03
Alternating attention (visual-spatial)						
Correct	.04	.70	−.01	−.28	−.18	−2.88*
RTs	−.16	−2.3**	−.02	−.39	.32	4.72**

*Note.* RT = Reading accuracy

* *p < *.05. **p < .001.

Attention skills were found to be useful predictors for all the dimensions of reading skill: “reading comprehension”: *F*(14) = 3.82, *p* < .001; reading accuracy”: *F*(14) = 4.63, *p* < .001; “reading speed” *F*(14) = 4.08, *p < *.001.

For “reading comprehension,” the *B* and *t* values showed the significant contribution to the model of the following predictors: “selective attention” (time) and visual-spatial “alternating attention” (response time). The “selective attention” scores were the better predictor for the “reading comprehension” scores; “selective attention”—times scores: *t*(272) = 3.28, *p < *.001; visual-spatial “alternating attention”—response time scores: *t*(272) = −3.22, *p* < .001.

With regard to reading accuracy, which was measured through “error rate” scores, the *B* and *t* values showed the significant contribution to the model of the following predictors: “auditory focused attention” error rate scores, *t*(272) = −3.68, *p* < .001; “selective attention” error rate scores, *t*(272) = −3.57 *p* < .001; “verbal alternating attention” error rate scores, *t*(272) = 2.75, *p* = .006; “distributed attention” error rate scores, *t*(272) = −2.07, *p* = .04; “visual focused attention” response time scores, *t*(272)* = *−2.21*, p* = .03; and “spatial focused attention” response time scores, *t*(272) = 2.07, *p* = . 03. With regard to the “reading speed,” the *B* and *t* values showed the significant contribution to the model of the following predictors: “selective attention,” error rate and response time scores: *t*(272) = −2.83, *p* = .02; *t*(272) = 2.43, *p* = .01, respectively; and “visual-spatial alternating attention,” *t*(272) = 4.72*, p* < .001.

### Descriptive and *t* Test Analysis of the Attention Scores by Good and Poor Readers

*T* test analyses for good and poor readers were calculated. Results showed that the “good readers” presented better performance in the “auditory focused attention,” *t*(286) = 2.18, *p* = .03, “visual-spatial focused attention,” *t*(286) = 4.90, *p* < .001, and “selective attention,” *t*(286) = −3.77, *p* < .001, tasks. Interestingly, the good readers were faster in their responses to all attentional tasks than the poor readers (see Table 3).

**Table 3.** Means, Standard Deviations, and *t* Test for All Measures by Good and Poor Readers.

Attention task	*df*	*M*	*SD*	*t*	*p*
Auditory focused attention	286				
Correct					
Good readers		8.76	.70	2.18	.030
Poor readers		8.50	1.26		
RTs					
Good readers		.732	.17	−2.34	.020
Poor readers		.78	.17		
Visual focused attention	286				
Correct					
Good readers		8.64	1.55	.73	.46
Poor readers		8.53	1.95		
RTs					
Good readers		.60	.09	−3.02	.003
Poor readers		.64	.16		
Visual-spatial attention	286				
Correct					
Good readers		11.49	.86	4.90	<.001
Poor readers		10.76	1.53		
RTs					
Good readers		.44	.10	−3.16	.002
Poor readers		.49	.13		
Selective attention	286				
Correct					
Good readers		27.01	4.56	3.77	<.001
Poor readers		24.61	6.12		
RTs					
Good readers		1.32	.24	−3.24	.001
Poor readers		1.63	1.12		
Alternating attention	286				
Correct(verbal)					
Good readers		7.36	2.20	.59	.556
Poor readers		7.22	1.99		
RTs (verbal)					
Good readers		56.63	21.85	−2.12	.034
Poor readers		63.62	32.86		
Correct (visual-spatial)					
Good readers		7.06	1.77	1.66	.097
Poor readers		6.67	2.10		
RTs (visual-spatial)					
Good readers		68.03	25.11	−3.85	<.001
Poor readers		80.79	30.79		
Distributed attention	286				
Correct					
Good readers		7.10	1.86	−.15	.875
Poor readers		7.13	1.87		
Good readers		.85	.14	−2.82	.005
Poor readers		.91	.22		

*Note. p *< .05.

## Discussion

The present study highlighted several interesting points concerning the relationships that occur among attention and reading skills in novice readers who spoke a transparent language. In particular, it contributed to clarifying the role of the main aspects of attention (“focused attention,” “selective attention,” “distributed attention,” and “alternating attention”) on the three dimensions (comprehension, accuracy, and speed) of reading skill.

Results showed that different aspects of attention influenced each dimension of reading skill. Moreover, reading ability was related to the correctness and rapidity of the attentional responses. Interestingly, not only the child’s ability to correctly focus visual stimuli but also the speed at which visual stimuli were focused influenced reading in novice readers. This result is of great interest and agrees with the results of the study of Serrano and Defior (2008), who suggested the possibility of phonological processing deficits in developmental dyslexic children who learn to read in Spanish, which is a transparent language like Italian. In this regard, Serrano and Defior (2008) found that children with dyslexia presented problems related to task performance time, and hypothesized that these difficulties might indicate an automatization-processing deficit. Although teachers rarely consider time in the assessment of reading skills, results of the present study confirm that the speed of decoding might be one of the main measures of reading difficulties in transparent languages (Serrano & Defior, 2008).

The results also showed that “selective attention” was the better predictor of “comprehension” skill. The capacity to resist the distraction of interfering stimuli was related to the ability to understand the meaning of text. The speed of shifting the attentional focus (“alternating attention”) also predicted “reading comprehension.” Probably, children who were able to shift focus rapidly scanned the different parts of the written text and elaborated the meaning of the written text easily.

“Auditory focused attention,” “distributed attention,” “selective attention,” “verbal alternating attention,” and the speed of focusing visual and visual-spatial stimuli influenced “reading accuracy,” which concerns the ability to decode text without mistakes. Although it is known that visual and visual-spatial attention are engaged at many levels of the process of recognizing printed word (e.g., McCandliss, Cohen, & Dehaene, 2003), literature in this field does not offer a clear and uncontroversial picture. In contrast to the studies that used the Stroop task, which highlighted the role of fully automatic processing on reading, several researchers found that the engagement of attention is a requirement to process visually presented words. In this regard, it is important to highlight that Stroop task may not be the best way to assess selective attention in children who are struggling to read words. It could be their poor reading ability rather than interference that slows them down.

Results of the present study showed that the ability to avoid interfering stimuli and the speed of focusing visual and visual-spatial stimuli contributed to reading accuracy, even in novice readers where reading is not an automated process. Moreover, the accuracy at which the “visual-spatial alternating attention” operated and the ability to rapidly avoid interfering stimuli influenced reading speed.

The analyses by good and poor readers showed that the good readers presented higher “auditory focused attention” scores than the poor readers. The role of auditory focused attention on reading skills was not surprising. It is well known that phonological skills influence learning to read (e.g., Miller & Kupfermann, 2009; Vellutino et al., 2004). Of interest, in the novice readers, the ability to correctly focus visual-spatial stimuli plays a higher role on reading performance than the ability to focus verbal stimuli. With respect to the reading acquisition, the capacity to discriminate the orientation of a stimulus and direct attention to specific stimuli is more important than the ability to recognize the letters. Often, kindergarten children can name the letters, but only the children who were able to focus the characteristics that differentiate the letters, such as the orientation to right, left, top, or down of the lines that compose them, are good readers.

## Conclusion

In conclusion, results showed that “selective attention,” which permits the avoidance of distraction, inhibiting the interference of environmental stimuli, predicted all the dimensions of reading skills. The others components of attention are diversely involved in reading and contribute significantly to reading acquisition.

Although this study has several limitations, because longitudinal data are not available, these findings may have educational and practical relevance. This study revealed the role of the different components of attention on reading acquisition; other recent findings showed that disruption of attentional mechanisms plays a causal role in reading disability (e.g., Luoni et al., 2015; Shaywitz & Shaywitz, 2008). For this reason, the early assessment of attention might contribute to reducing reading difficulties when children begin primary school. The investigation of the role of the main components of attention on reading skill might favor the development of new strategies of intervention in dyslexic children and in children at risk of developing learning difficulties. Attention can be measured easily in school. Its measurement could allow us to identify those children who could benefit from attention training, with the aim to facilitate reading acquisition. Increasing attention skills could produce positive effects on the acquisition of the basic scholastic skills, such as reading, reducing the risk of developing learning difficulties.

## Notes

1 In the new norms of the “Speed and Accuracy” test of the reading battery, the authors excluded the outliers. The authors also noted that in many samples some children present reading scores particularly low (Cornoldi et al., 2010). In this research, the outliers were not excluded because they represented a genuine value of the population. For this reason, and for the characteristics of the Italian scholastic system, in which preschool is not compulsory and children who begin school present different levels of school readiness, in this sample, numerous children were classified as poor children and obtained reading scores lower than that correspondent to the 15th percentile.

## References

[bibr1-2041669517718557] Akhtar N. Enns J. T. (1989) Relations between covert orienting and filtering in the development of visual attention. Journal of Experimental Child Psychology 48: 315–334. 2794859 10.1016/0022-0965(89)90008-8

[bibr2-2041669517718557] Baddeley A. (1982) Reading and working memory. Bulletin of the British Psychological Society 35: 414–417. 12264587

[bibr3-2041669517718557] Bar-Kochva I. (2013) What are the underlying skills of silent reading acquisition? A developmental study from kindergarten to the 2nd grade. Reading and Writing 26: 1417–1436.

[bibr4-2041669517718557] Bishop, D. V. M., & Snowling, M. (2004). Developmental dyslexia and specific language impairment: Same or different? *Psychological Bulletin, 130*, *6*, 858–886. 10.1037/0033-2909.130.6.858 15535741

[bibr5-2041669517718557] Bosse M. L. Tainturier M.-J. Valdois S. (2007) Developmental dyslexia: The visual attention span deficit hypothesis. Cognition 104: 2, 198–230. 10.1016/j.cognition.2006.05.009 16859667

[bibr6-2041669517718557] Bosse M. L. Vadois S. (2009) Influence of the visual attention span on child reading performance: Cross-sectional study. Journal of Research in Reading 32: 230–253.

[bibr7-2041669517718557] Brodeur D. A. Trick L. M. Enns J. T. (1997) Selective attention over the lifespan. In: Burack J. A. Enns J. T. (eds) Attention, development, and psychopathology: A merging of disciplines, New York, NY: Guilford Press, pp. 74–94.

[bibr8-2041669517718557] Braun J. (1998) Divided attention: Narrowing the gap between brain and behavior. In: Parasuraman R. (ed.) The attentive brain, Cambridge: MIT Press, pp. 327–351.

[bibr9-2041669517718557] Brook U. Boaz M. (2005) Attention deficit and hyperactivity disorder (ADHD) and learning disabilities (LD): Adolescents perspective. Patient Education and Counselling 58: 187–191. 10.1016/j.pec.2004.08.011 16009295

[bibr10-2041669517718557] Bunge S. A. Dudukovic N. M. Thomason M. E. Vaidya C. J. Gabrieli J. D. (2002) Immature frontal lobe contributions to cognitive control in children: Evidence form fMRI. Neuron 17: 33, 301–311. 10.1016/s0896-6273(01)00583-9 PMC4535916 11804576

[bibr11-2041669517718557] Cannavò R. Conti D. Di Nuovo A. (2016) Computer-aided assessment of aviation pilots attention: Design of an integrated test and its empirical validation. Applied Computing and Informatics 12: 16–26.

[bibr12-2041669517718557] Castles A. Coltheart M. (2004) Is there a causal link from phonological awareness to success in learning to read? Cognition 91: 77–111. 14711492 10.1016/s0010-0277(03)00164-1

[bibr13-2041669517718557] Chan R. K. Shum D. Toulopoulou T. Chen E. Y. H. (2008) Assessment of executive functions: Review of instruments and identification of critical issues. Archives of Clinical Neuropsychology 23: 201–216. 18096360 10.1016/j.acn.2007.08.010

[bibr15-2041669517718557] Colombo J. (2001) The development of visual attention in infancy. Annual Review of Psychology 52: 337–367. 10.1146/annurev.psych.52.1.337 11148309

[bibr16-2041669517718557] Commodari E. (2012) Attention skills and risk of developing learning difficulties. Current Psychology 31: 17–34.

[bibr17-2041669517718557] Commodari E. (2016) Voluntary visual orienting in schoolchildren: How visual orienting skills change during primary school voluntary visual orienting in schoolchildren. Perceptual and Motor skills 122: 988–1001. 27226225 10.1177/0031512516652034

[bibr18-2041669517718557] Commodari E. Di Blasi M. (2014) The role of the different components of attention on calculation skills. Learning and Individual Differences 32: 225–232.

[bibr19-2041669517718557] Commodari E. Guarnera M. (2005) Attention and reading skills. Perceptual and Motor Skills 100: 3753–3786. 10.2466/pms.100.2.375-386 15974348

[bibr20-2041669517718557] Cornoldi C. Colpo G. (2011) New reading battery MT 2 for primary school. Nuove prove di lettura MT 2 per la scuola elementare, Firenze: Organizzazioni Speciali.

[bibr21-2041669517718557] Cornoldi C. Colpo G. Gruppo M T (1998) Nuove prove di lettura MT per la scuola elementare, Firenze: Organizzazioni Speciali.

[bibr22-2041669517718557] Cornoldi C. Tressoldi P. Perini N. (2010) Assessment of reading speed and accuracy in decoding text. New norms and some clarifications. Valutare la rapidità e la correttezza della lettura di brani. Nuove norme e alcune chiarificazioni per l’uso delle prove MT. *Dislessia* 7: 89–100.

[bibr24-2041669517718557] Defior S. (2004) Phonological awareness and learning to read: A crosslinguistic perspective. In: Bryant P. Nunes T. (eds) Handbook on children’s literacy, Dordrecht, Netherlands: Kluwer, pp. 163–174.

[bibr25-2041669517718557] Di Nuovo S. (2006) Assesment of attention: from sperimental research to application. La valutazione dell’attenzione: dalla ricerca sperimentale ai contesti applicativi, Milano: Franco Angeli.

[bibr26-2041669517718557] Dye M. W. G. Bavelier D. (2009) Differential development of visual attention skills in school-age children. Vision Research 50: 52–459. 10.1016/j.visres.2009.10.010 PMC2824025 19836409

[bibr27-2041669517718557] Enns J. T. (1990) The development of attention: Research and theory, Amsterdam, Netherlands: North-Holland.

[bibr28-2041669517718557] Ferretti G. Mazzotta S. Brizzolara D. (2008) Visual scanning and reading ability in normal and dyslexic children. Behavioural Neurology 9: 87–92. 10.1155/2008/564561 PMC5452447 18413924

[bibr29-2041669517718557] Franceschini S. Gori S. Ruffino M. Pedrolli K. Facoetti A. (2012) A causal link between visual spatial attention and reading acquisition. Current Biology 22: 814–819. 22483940 10.1016/j.cub.2012.03.013

[bibr30-2041669517718557] Francis A. L. Kaganovich N. Driscoll-Huber C. (2008) Cue-specific effects of categorization training on the relative weighting of acoustic cues to consonant voicing in English. Journal of the Acoustical Society of America 124: 1234–1251. 18681610 10.1121/1.2945161 PMC2680590

[bibr31-2041669517718557] Gerardi-Caulton G. (2000) Sensitivity to spatial conflict and the development of self-regulation in children 24–36 months of age. Developmental Science 3: 397–404.

[bibr32-2041669517718557] Gordon P. C. Eberhardt J. L. Rueckl J. G. (1993) Attentional modulation of the phonetic significance of acoustic cues. Cognitive Psychology 25: 1–42. 8425384 10.1006/cogp.1993.1001

[bibr33-2041669517718557] Guarnera M. Commodari E. Peluso C. (2013) Rotation and generation of mental imagery in children with specific language impairment. Acta Paediatrica 102: 539–543. 23350585 10.1111/apa.12162

[bibr34-2041669517718557] Hahn B. Wolkenberg F. A. Thomas J. Ross T. J. Myers C. S. Heishman S. J. Stein E. A. (2008) Divided versus selective attention: Evidence for common processing mechanisms. Brain Research 18: 137–146. 10.1016/j.brainres.2008.03.058 PMC2497334 18479670

[bibr35-2041669517718557] Hawelka S. Wimmer H. (2005) Impaired visual processing of multi-element arrays is associated with increased number of eye movements in dyslexic reading. Vision Research 45: 855–863. 15644226 10.1016/j.visres.2004.10.007

[bibr36-2041669517718557] Hoffman J. E. Subrananian B. (1995) The role of visual attention in saccadic eye movements. Perception & Psychophysics 57: 787–795. 7651803 10.3758/bf03206794

[bibr37-2041669517718557] Keith R. W. (1994) Auditory Continuous Performance Test (ACPT), London, England: Harcourt Brace Educational Measurement.

[bibr38-2041669517718557] Kristja’nsson A. (2007) Saccade landing point selection and the competition account of pro- and antisaccade generation: The involvement of visual attention—a review. Scandinavian Journal of Psychology 48: 97–113. 17430363 10.1111/j.1467-9450.2007.00537.x

[bibr39-2041669517718557] Kristjánsson A. (2011) The intriguing interactive relationship between visual attention and saccadic eye movements. In: Liversedge L. Gilchrist I. D. Everling S. (eds) Oxford handbook of eye movements, Oxford, England: Oxford University Press, pp. 455–470.

[bibr40-2041669517718557] La Berge D. Samuels S. J. (1974) Toward a theory of automatic information processing in reading. Cognitive Psychology 6: 293–323.

[bibr41-2041669517718557] Lezak M. D. Howieson D. B. Bigler E. D. Trane D. (2015) Neuropsychological assessment, Oxford, NY: Oxford University Press.

[bibr42-2041669517718557] Luoni C. Balottin U. Zaccagnino M. Brembilla L. Livetti G. Termine C. (2015) Reading difficulties and attention-deficit/hyperactivity behaviours: Evidence of an early association in a nonclinical sample. Journal of Research in Reading 38: 73–90.

[bibr43-2041669517718557] McCandliss B. D. Cohen L. Dehaene S. (2003) The visual word form area: Expertise for reading in the fusiform gyrus. Trends of Cognitive Science 7: 293–299. 10.1016/s1364-6613(03)00134-7 12860187

[bibr44-2041669517718557] Mezzacappa E. (2004) Alerting, orienting and executive attention. Developmental properties and sociodemographic correlated in and epidemiological sample of young, urban children. Child Development 75: 1373–1386. 15369520 10.1111/j.1467-8624.2004.00746.x

[bibr45-2041669517718557] Miller P. Kupfermann A. (2009) The role of visual and phonological representations in the processing of written words by readers with diagnosed dyslexia: Evidence from a working memory task. Annual of Dyslexia 59: 12–33. 10.1007/s11881-009-0021-1 19308736

[bibr46-2041669517718557] Muter V. Hulme C. Snowling M. J. Stevenson J. (2004) Phonemes, rimes and language skills as foundations of early reading development: Evidence from a longitudinal study. Developmental Psychology 40: 665–681. 15355157 10.1037/0012-1649.40.5.665

[bibr47-2041669517718557] Pradhan B. Nagendra H. R. (2008) Normative data for letter-cancellation tasks in school children. International Journal of Yoga 1: 72–75. 21829288 10.4103/0973-6131.43544 PMC3144614

[bibr48-2041669517718557] Pammer K. Lavis R. Cooper C. Hansen P. C. Cornelissen P. L. (2005) Symbol-string sensitivity and adult performance in lexical decision. Brain Language 94: 278–296. 16098378 10.1016/j.bandl.2005.01.004

[bibr49-2041669517718557] Pammer K. Lavis R. Hansen P. Cornelissen P. L. (2004) Symbol-string sensitivity and children’s reading. Brain Language 89: 601–610. 15120551 10.1016/j.bandl.2004.01.009

[bibr50-2041669517718557] Parasuraman R. (1998) The attentive brain: Issues and prospects. In: Parasuraman R. (ed.) The attentive brain, Cambridge: MIT Press, pp. 3–15.

[bibr51-2041669517718557] Pelli D. G. Burns C. W. Farell B. Moore-Page D. C. (2006) Feature detection and letter identification. Vision Research 46: 46–74. 10.1016/j.visres.2006.04.023 16808957

[bibr52-2041669517718557] Peterson S. E. Posner M. I. (1990) The attention system of the human brain. Annual Review of Neurosciences 13: 25–42. 10.1146/annurev.ne.13.030190.000325 2183676

[bibr53-2041669517718557] Peterson S. E. Posner M. I. (2012) The attention system of the human brain: 20 years after. Annual Review of Neuroscience 35: 75–89. 10.1146/annurev-neuro-062111-150525 PMC3413263 22524787

[bibr54-2041669517718557] Posner M. I. (2012) Attentional networks and consciousness. Frontiers of Psychology 3: 64. 10.3389/fpsyg.2012.00064 PMC3298960 22416239

[bibr55-2041669517718557] Posner M. I. Petersen S. E. Fox P. T. Raichle M. E. (1988) Localization of cognitive operations in the human brain. Science 240: 1627–1631. 3289116 10.1126/science.3289116

[bibr56-2041669517718557] Posner M. I. Raichle M. E. (1994) Images of mind, New York, NY: Scientific American Library.

[bibr57-2041669517718557] Posner M. I. Rueda M. R. Kanske P. (2007) Probing the mechanisms of attention. In: Cacioppo J. T. Tassinary J. G. Berntson G. G. (eds) Handbook of psychophysiology, 3rd ed Cambridge, England: Cambridge University Press, pp. 410–432.

[bibr58-2041669517718557] Posner M. I. Rothbart M. K. Sheese B. S. Voelker P. (2014) Developing attention: Behavioral and brain mechanisms. Advances in Neuroscience 2014: 405094. 25110757 10.1155/2014/405094 PMC4125572

[bibr59-2041669517718557] Pozuelos J. P. Paz-Alonso P. M. Castillo A. Fuentes L. J. Rueda M. R. (2014) Development of attention networks and their interactions in childhood. Developmental Psychology 50: 2405–2415. 25069052 10.1037/a0037469

[bibr60-2041669517718557] Reynolds M. Besner D. (2006) Reading aloud is not automatic: Processing capacity is required to generate a phonological code from print. Journal of Experimental Human Perception and Performance 32: 1303–1323. 10.1037/0096-1523.32.6.1303 17154774

[bibr61-2041669517718557] Ridderinkhoff K. R. van der Molen M. W. Band G. P. Bashore T. R. (1997) Sources of interference from irrelevant information: A developmental study. Journal of Experimental Child Psychology 65: 315–341. 9178963 10.1006/jecp.1997.2367

[bibr62-2041669517718557] Rueda, M. R. (2014). Development of attention. In K. N. Ochsner, & S. Kosslyn (Eds.), *Oxford handbook of cognitive neuroscience*, *1*, 296–318.

[bibr63-2041669517718557] Rueda M. R. Fan J. McCandliss B. D. Halparin J. D. Gruber D. B. Lercari L. P. Posner M. I. (2004) Development of attentional networks during childhood. Neuropsychologia 42: 1029–1040. 15093142 10.1016/j.neuropsychologia.2003.12.012

[bibr64-2041669517718557] Rueda M. R. Posner M. I. Rothbart M. K. (2005) The development of executive attention: Contributions to the emergence of Self-Regulation. Developmental Neuropsychology 28: 573–594. 16144428 10.1207/s15326942dn2802_2

[bibr65-2041669517718557] Ruffino M. Trussardi A. M. Gori S. Finzi A. Giovagnoli S. Menghini D. Facoetti A. (2010) Attentional engagement deficits in dyslexic children. Neuropsychologia 48: 3793–3801. 20833191 10.1016/j.neuropsychologia.2010.09.002

[bibr66-2041669517718557] Salthouse T. A. Fristoe N. M. Uneweaver T. A. Coon V. E. (1995) Aging of attention: Does the ability to divide decline? Memory and Aging 23: 59–71. 10.3758/bf03210557 7885266

[bibr67-2041669517718557] Samuels S. J. (1987) Information processing abilities and reading. Journal of Learning Disability 20: 18–22. 10.1177/002221948702000104 3805887

[bibr68-2041669517718557] Savage R. Cornish K. Manly T. Hollis C. (2006) Cognitive processes in children's reading and attention: The role of working memory, divided attention, and response inhibition. British Journal of Psychology 97: 365–385. 16848949 10.1348/000712605X81370

[bibr69-2041669517718557] Sears C. R. Pylyshyn Z. W. (2000) Multiple object tracking and attentional processing. Canadian Journal of Experimental Psychology 54: 1–14. 10721235 10.1037/h0087326

[bibr70-2041669517718557] Serrano F. Defior S. (2008) Dyslexia speed problems in a transparent orthography. Annales of Dyslexia 58: 81–95. 10.1007/s11881-008-0013-6 18483868

[bibr71-2041669517718557] Seymour P. H. Aro M. Erskine J. M. (2003) Foundation literacy acquisition in European orthographies. British Journal of Psychology 94: 143–174. 12803812 10.1348/000712603321661859

[bibr72-2041669517718557] Share D. L. (1995) Phonological recoding and self-teaching: Sine qua non of reading acquisition. Cognition 55: 151–218. 7789090 10.1016/0010-0277(94)00645-2

[bibr73-2041669517718557] Share D. L. (2004) Orthographic learning at a glance: On the time course and developmental onset of self-teaching. Journal of Experimental Child Psychology 87: 267–298. 15050455 10.1016/j.jecp.2004.01.001

[bibr74-2041669517718557] Shaywitz S. E. Shaywitz B. A. (2008) Paying attention to reading: The neurobiology of reading and dyslexia. Developmental Psychopathology 20: 1329–1349. 10.1017/S0954579408000631 18838044

[bibr75-2041669517718557] Sohlberg M. M. Mather C. A. (1989) Introduction to cognitive rehabilitation, New York, NY: Guildford Press.

[bibr76-2041669517718557] Stern P. Shalev L. (2013) The role of sustained attention and display medium in reading comprehension among adolescents with ADHD and without it. Research in Developmental Disabilities 34: 431–439. 23023301 10.1016/j.ridd.2012.08.021

[bibr77-2041669517718557] Stroop J. R. (1935) Studies of interference in serial verbal reactions. Journal of Experimental Psychology 6: 224–243.

[bibr78-2041669517718557] Schul R. Townsend J. Stiles J. (2003) The development of attentional orienting during the school-age years. Developmental Science 6: 262–272.

[bibr79-2041669517718557] Tirosh E. Cohen A. Berger J. Davidovitch M. Cohen-Ophir M. (2001) Neurodevelopmental and behavioural characteristics in learning disabilities and attention deficit disorder. European Journal of Pediatric Neurology 5: 253–258. 10.1053/ejpn.2001.0525 11764183

[bibr80-2041669517718557] Trick L. M. Enns J. T. (1998) Lifespan changes in attention: The visual search task. Cognitive Development 13: 369–386.

[bibr81-2041669517718557] Valdois S. Bosse M. L. Tainturier M. J. (2004) The cognitive deficits responsible for developmental dyslexia: Review of evidence for a selective visual attentional disorder. Dyslexia 10: 339–363. 15573964 10.1002/dys.284

[bibr82-2041669517718557] Van den Boer M. van Bergen E. de Jong P. F. (2014) Underlying skills of oral and silent reading. Journal of Experimental Child Psychology 128: 138–151. 25173643 10.1016/j.jecp.2014.07.008

[bibr83-2041669517718557] Van den Boer M. de Jong P. F. Haentjens-van Meeteren M. M. (2013) Modeling the length effect: Specifying the relation with visual and phonological correlates of reading. Scientific Studies of Reading 17: 243–256.

[bibr84-2041669517718557] Vellutino F. R. Fletcher J. M. Snowling M. J. Scanlon D. M. (2004) Specific reading disabilities (dyslexia): What have we learned in the past four decades? Journal of Child Psychology and Psychiatry 45: 40137–40148. 10.1046/j.0021-9630.2003.00305.x 14959801

[bibr85-2041669517718557] Vellutino F. R. Tunmer W. E. Jaccard J. J. Chen R. (2007) Components of reading ability: Multivariate evidence for a convergent skills model of reading development. Scientific Studies of Reading 11: 3–32.

[bibr186-2041669517718557] Waszak, F., Li, S.-C., & Hommel, B. (2010). The development of attentional networks: cross-sectional findings from a life span sample. *Developmental Psychology*, *46*, 337–349. 10.1037/a0018541 20210494

[bibr86-2041669517718557] Yang S. Yang H. (2016) Bilingual effects on deployment of the attention system in linguistically and culturally homogenous children and adults. Journal of Experimental Child Psychology 146: 121–136. 26930166 10.1016/j.jecp.2016.01.011

